# Association of Fall-Risk Factors and Margin of Stability While Tripping in Community-Dwelling Older Adults: Experimental Pilot Study

**DOI:** 10.2196/74418

**Published:** 2026-02-05

**Authors:** Kim Sarah Sczuka, Marc Schneider, Ngaire Kerse, Clemens Becker, Jochen Klenk

**Affiliations:** 1 Department of Clinical Gerontology, Robert-Bosch-Hospital, Auerbachstraße 110, Stuttgart, 70376, Germany, +49 7118101 ext 6070; 2 Department of General Practice and Primary Health Care, University of Auckland, Auckland, New Zealand; 3 Digital Geriatric Medicine, Medical Clinic, Heidelberg University, Heidelberg, Germany; 4 Institute of Epidemiology and Medical Biometry, Ulm University, Ulm, Germany; 5 IB University of Health and Social Sciences, Study Center Stuttgart, Stuttgart, Germany

**Keywords:** dynamic balance, fall-related activity, fall risk, fall risk factor, laboratory setting, margin of stability

## Abstract

**Background:**

Falls are a leading cause of injury among older adults, often resulting from dynamic balance disturbances. It is influenced by a complex interplay of intrinsic and extrinsic fall-risk factors. To identify individual fall risks, it is important to understand the underlying associations.

**Objective:**

This study aimed to build an experimental setup modeling selected factors leading to a loss of balance, measured by the margin of stability (MoS) in an ecologically valid real-world example (tripping). Additionally, these analyses aimed to assess the feasibility and safety of the protocol and to explore the use of the MoS as part of a prototypical dynamic fall-risk model to differentiate between fall-risk groups.

**Methods:**

Nineteen community-dwelling older adults (mean age of 71, SD 3.67 y; n=7, 37% women) completed the tripping protocol involving perturbations under various conditions. Clinical assessments were used to identify relevant fall-related intrinsic fall-risk factors. MoS was measured using an 8-camera motion capture system. Receiver operating characteristic analyses determined the ability of MoS to distinguish between low and high fall-risk groups.

**Results:**

Approximately one-quarter of participants discontinued before or at the start of the tripping scenario because of discomfort or fear of perturbations, indicating that perceived safety is an important feasibility factor. Perturbations significantly disrupted MoS, with a median MoS of −106.05 (IQR –181.40 to –41.50) mm during the perturbed step compared to 114 (IQR 81.20-155.20) mm in the preperturbation step. Recovery steps showed progressive stabilization, with the second recovery step achieving a median MoS of 88.45 (IQR 47.50-137.80) mm. The second recovery step exhibited the highest predictive accuracy for fall-risk differentiation, with area under the curve values reaching 82.3% during slow walking with a series of right-sided perturbations. In contrast, fast walking with random perturbations yielded lower area under the curve values (64.9%). Slow walking conditions generally demonstrated the clearest separation between fall-risk groups.

**Conclusions:**

This pilot and feasibility study demonstrates the applicability of a tripping paradigm to perturb MoS in older adults and provides preliminary insights into its association with fall-risk indices. While the protocol proved safe and feasible for fit older adults, perceived safety limited full participation. The findings are exploratory and intended to guide the design of larger prospective studies rather than to establish predictive conclusions. These data suggest that MoS during controlled tripping may help differentiate fall-risk strata, but confirmation will require adequately powered studies in more diverse and frailer older populations—and across multiple real-world scenarios—before any clinical implementation can be considered.

## Introduction

Falls are a major public health concern among older adults, with significant implications for morbidity, disability, and long-term care. According to the World Health Organization, falls are the second leading cause of unintentional injury deaths worldwide, with an estimated 684,000 fatal falls each year—most of them among individuals aged over 60 years [1]. Furthermore, approximately 28% to 35% of people aged 65 years and older fall each year, and recent systematic reviews confirm that physical risk factors such as gait and balance impairments, frailty, chronic disease, and psychological conditions (eg, fear of falling) are among the most consistently associated with fall risk [2,3]. Falls can result in serious consequences including fractures, reduced mobility, loss of independence, and institutionalization. Consequently, research on falls and fall-related aspects has intensified in recent years. This highlights the urgent need for effective prevention strategies.

Despite numerous prevention efforts, fall risk in older adults remains difficult to manage due to the complex interplay of physiological, behavioral, and environmental factors. Systematic reviews demonstrate that multifactorial and exercise-based interventions can reduce fall rates, but findings remain heterogeneous, and many programs show limited transferability to real-world contexts [4-7]. Many current approaches fail to adequately account for this dynamic and multifactorial nature, limiting their effectiveness in real-life contexts [2]. Traditional assessments, such as binary outcomes (fall or no fall), provide only limited insights into subtle changes in fall risk. To overcome these limitations, continuous biomechanical measures like the margin of stability (MoS) have emerged as promising tools for assessing instantaneous fall risk. The MoS, introduced by Hof et al [8], quantifies dynamic stability by considering both the position and velocity of the center of mass (CoM) relative to the base of support (BoS). While MoS has become a widely accepted measure of dynamic stability, most applications to date have been limited to controlled laboratory conditions, such as steady-state treadmill walking. Reviews emphasize that this restricts ecological validity, as real-world falls typically involve unexpected perturbations rather than regular gait cycles [9-11]. Biomechanically, a system is stable when the projected CoM is within the BoS boundaries. However, dynamic situations, such as walking or perturbations, require extending this static concept. The MoS calculates the distance between the extrapolated CoM and the BoS, incorporating movement dynamics. If the extrapolated CoM surpasses the BoS, the MoS becomes negative, indicating instability and the need for corrective actions to prevent a fall. Populations at risk, such as stroke survivors or amputees, often adopt compensatory strategies to increase their MoS. These include widening step width to enhance mediolateral stability, and in some cases, reducing step length to improve anteroposterior MoS [12-14]. These adaptations may help mitigate instability in the frontal plane during walking. While these adaptations enhance stability in the short term, they may limit the ability to respond effectively to unexpected perturbations [15]. Moreover, age-related compensatory strategies such as increased mediolateral MoS have also been observed in healthy older adults, suggesting an altered motor control mechanism to preserve balance [16]. However, these compensatory strategies may mask deficits under steady-state conditions. Studies have consistently shown that when older adults are exposed to perturbations such as slips or trips, they often demonstrate a reduced ability to restore a safe MoS, which is strongly linked to the increased fall risk [11,17,18]. Likewise, several investigations confirmed that reduced MoS during perturbations is associated with impaired balance recovery and elevated fall risk [19-23]. Additionally, Robinovitch et al [18] highlighted from real-world fall video analyses that overconfidence and incorrect weight shifts are major contributors to falls in older adults. These findings emphasize the need to examine both biomechanical and behavioral aspects of fall risk. Against this background, perturbation paradigms such as tripping provide ecologically valid insights into stability and recovery, complementing traditional steady-state gait assessments. To investigate the dynamic interplay of intrinsic and extrinsic factors contributing to falls, this study builds upon findings from the FARSEEING project. The FARSEEING consortium, funded by the EU’s 7th Framework Program for Research, collected a dataset of over 200 validated real-world falls measured by body-worn sensors, providing insights into common fall scenarios and environmental factors [24]. The analysis of these real-world data highlighted tripping as one of the most frequent and ecologically valid fall scenarios, which forms the basis for the experimental setup in this study.

However, most sensor devices lack context, environmental conditions, and intrinsic fall risk factors. Reenactment of known fall events has proven to be an effective method for capturing the complexity of real-world falls in a safe, repeatable environment [25]. This method improves simulation protocols and produces realistic fall simulations [26] while enabling further analysis.

In addition, predictive models such as the web-based Fall Risk Assessment Tool for community-dwelling older people (FRAT-up) tool [27] can complement experimental approaches by integrating clinical and environmental factors that contribute to fall risk.

Together, these methodological advances highlight the importance of combining experimental, biomechanical, and predictive approaches to better understand the multifactorial nature of falls. To address this complexity, it is essential to investigate scenarios that are both ecologically valid and relevant to real-life situations. Tripping, being one of the most common and significant causes of falls, serves as an ideal experimental paradigm because of its practical relevance and the ability to replicate it under controlled conditions. This study is part of a broader protocol encompassing four fall-related scenarios aimed at capturing the multifactorial nature of falls in older adults [28]. Tripping was chosen as the initial focus due to its prevalence and ecological validity, providing a solid foundation for exploring dynamic stability in hazardous situations.

Accordingly, the present pilot and feasibility study had two objectives: first, to examine the feasibility and construct validity of a laboratory-based tripping paradigm designed to perturb the MoS in community-dwelling older adults; and second, to explore preliminary associations between MoS parameters during perturbation and recovery steps and an independently derived fall-risk index.

## Methods

### Ethical Considerations

This study was approved by the ethics committee of the University of Tübingen (protocol: 245/2018BO2) and forms part of a broader registered protocol on fall-related scenarios. All participants provided written informed consent for study participation and for the use of deidentified data for scientific purposes. Data were pseudonymized using numeric identifiers and stored on secure institutional servers with access restricted to the study team. No video recordings obtained during the study are published in this paper or supplementary material. No images included in this study permit participant identification. Participants were offered parking tickets to cover travel expenses if needed.

### Study Design

This analysis, as a focused part of a broader experimental pilot study on perturbations, simulated a tripping scenario with task variations that increased complexity, such as carrying an object or performing a cognitive task. The broader study also included other perturbation scenarios, such as opening a door, walking on a slippery surface, and standing in a bus while it accelerates. All participants underwent extensive clinical and quantitative assessments and performed the full study protocol to evaluate the effect of different fall scenarios on individual fall risk at the Robert Bosch Hospital, Stuttgart.

### Eligibility Criteria

German-speaking, community-dwelling, healthy, as well as prefrail individuals aged 60 years or older were recruited. People who needed more than 12 seconds for the Timed Up-and-Go (TUG) test [29] were classified as prefrail. Exclusion criteria included a current diagnosis of a serious neurological or sensory disease (eg, Parkinson disease and a history of stroke); uncorrected visual difficulties; dizziness; uncontrolled or serious cardiovascular or metabolic disorders (eg, diabetes with neuropathy); cognitive impairments beyond dementia that would preclude informed consent or following multistep instructions (eg, moderate to severe mild cognitive impairment and psychiatric conditions interfering with task compliance) as well as contraindications for perturbations, including osteoporosis, joint replacement, joint stiffening, severe spinal disease, or recent fracture. Participants had to be able to walk continuously for at least 5 minutes without walking aids. This threshold was chosen to ensure that participants could safely complete repeated treadmill trials, adapt to the treadmill, and tolerate perturbations without excessive fatigue. All participants provided their written informed consent and were insured.

### Recruitment

Participants were community-dwelling older adults who had previously indicated willingness to be contacted for further research after taking part in earlier gerontological studies at the Robert Bosch Hospital, Stuttgart, Germany. Potential participants were first contacted either by telephone or—if initial information was provided by email or postal mail—were subsequently followed up by telephone. During this call, the study objectives and procedures were explained, allowing individuals to decide whether they were interested in attending an appointment at the hospital’s gait laboratory. At the appointment, participants received detailed written study information and had the opportunity to discuss any questions before providing written informed consent.

The study sessions took place in the gait laboratory of the Department of Geriatric Rehabilitation, equipped with an instrumented treadmill and safety harness system. In total, 26 individuals were invited to participate. One individual was excluded on arrival because the inclusion criterion of being able to walk freely for at least 5 minutes was not met, and therefore, no measurements were initiated. Of the remaining 25 participants who started the protocol, 19 completed the full tripping scenario and were included in the analysis. The main reasons for noncompletion were withdrawal due to discomfort or fear of perturbations.

In line with the recommendations by Thabane et al [30], the sample size was set pragmatically based on feasibility considerations. As the primary goal was not hypothesis testing but to assess recruitment potential, study procedures, and the need for protocol adaptation, no formal sample size calculation was conducted.

### Clinical Assessments

To identify relevant intrinsic fall risk factors and to describe the cohort, appropriate clinical assessments were selected. These clinical assessments were conducted for all participants prior to the experimental protocol, and all assessments were performed by trained assessors.

Clinical data included sex, age, body weight, physical activity, and medical history. Additionally, the general state of health and comorbidity were evaluated using the Functional Comorbidity Index (range 0‐18, higher scores indicating more comorbidities) [31]. The occurrence of falls over the past 12 months was recorded. Fall-related self-efficacy was assessed using the short form of the Falls Efficacy Scale-International (FES-I; range 7‐28, higher scores reflecting greater concern about falling), which is strongly correlated with both previous and subsequent falls [32].

Physical performance was evaluated using the Short Physical Performance Battery (SPPB; range 0‐12, higher scores indicating better performance) [33] and the TUG test (time in s to stand up, walk 3 m, turn, return, and sit down again; longer times reflect lower mobility) [29].

### Experimental Setup

All participants followed the same overarching study protocol, which included 4 ecologically valid scenarios: tripping, opening a door, walking on a slippery surface, and standing in a bus while it accelerates. These scenarios were selected based on data from the FARSEEING database [24], with the tripping scenario standing out due to its high frequency and relevance in real-life fall situations, as well as its significant public health impact. Consequently, this first analysis focuses specifically on the tripping scenario. For safety reasons and to prevent injuries, participants wore a safety harness that ensured no contact with the ground except for their feet. All study participants received instructions for safe performance (including guidance on shoes and safety harness) and were asked to perform all paradigms as long as they felt comfortable. The test protocol involved the primary activity of tripping while walking on a treadmill, combined with additional tasks to progressively increase the level of difficulty.

To induce tripping repeatedly, 2 attachment loops were fixed to the participants’ heels with a custom-made strap fixation (see Multimedia Appendix 1 for a schematic illustration of the experimental setup). Initially, participants walked on the treadmill at a self-selected gait speed to acclimate to the treadmill. Self-selected speed was defined as a comfortable walking pace at which participants reported feeling at ease on the treadmill. They were informed that the speed would later be increased, so they were asked to choose a relaxed, “strolling” pace rather than a fast or strenuous one. After this habituation phase, the inelastic cords were hooked into the attachment loops. Two inelastic cords, attached behind the treadmill, were adjusted in length with low resistance via a coil spring while the participant walked. The length variation of the cords could be interrupted at the push of a button. When the button was pressed at the end of the stance phase, the subsequent swing phase was perturbed. At random intervals, participants’ feet were briefly blocked until the button was released, perturbing the swing phase while allowing continuous walking without stopping or limping. The sequence of perturbations was pseudorandomized: one random sequence was generated at the beginning of the study and then applied identically to all participants. This sequence was unknown to the participants and consisted of 4 right-foot and 3 left-foot perturbations. A fall event was defined by registering more than 30% of body weight on a load cell attached to the safety harness [34].

Additional tasks included carrying a laundry basket, interacting with a memory buzzer game, and increasing the self-selected gait speed by 33%. The memory buzzer was a children’s electronic memory game with 1 central button and 4 colored outer buttons that lit up and were accompanied by an acoustic signal. Participants were required to press the correct button as quickly as possible. If successful, the sequence was extended by 1 additional stimulus, while the previous sequence had to be repeated in the correct order. This created a progressively more demanding cognitive motor dual-task condition during treadmill walking.

### Measurement Instruments

An 8-camera VICON T10 system (Vicon Motion Systems Ltd) was used to record absolute body motions and limits of stability at 100 Hz. The VICON motion tracking system is able to measure a person’s movement and its duration with high precision. Reflective markers were placed at anatomical landmarks (eg, shoulders, pelvis, knees, and ankles) according to the standard plug-in gait model (Vicon, Oxford Metrics). This widely used biomechanical model estimates joint centers and whole-body CoM from segmental anthropometric data, allowing reproducible calculation of kinematic variables. This means that the software reconstructs a simplified skeleton from the marker positions and uses it to calculate gait parameters such as CoM position and velocity. Treadmill speed was continuously measured via the same system.

All laboratory experiments in this project were also recorded by a video camera (Basler pilot piA640-210gc).

### Outcome Measure and Data Analysis

3D data were processed in Vicon Nexus 2.14.1 and analyzed in MATLAB (version r2023a; the MathWorks Inc). Small gaps in marker trajectories, up to 20 frames, were filled with a spline function, and marker trajectories were filtered with the Vicon plug-in gait standard Woltring filter [35]. Large gaps were filled using the Vicon Bodybuilder. The marker-based CoM position was extracted from the plug-in gait model. Using a custom-developed algorithm in MATLAB, toe-off and heel strike events were automatically detected. During perturbations, events were manually set if the automatic algorithm missed events due to transient marker occlusions. In such cases, synchronized video and marker trajectories were reviewed frame-by-frame. This ensured consistent event identification despite occasional signal artifacts.

Once the events were defined, the MoS was calculated for each heel strike in the anterior-posterior direction. The calculation was based on the approach described by Hof et al [8], but with modifications to account for treadmill conditions. Specifically, the extrapolated center of mass (*x*_CoM_) was computed by incorporating treadmill belt speed into the CoM velocity (sign chosen according to the laboratory axis conventions):


$$x_{\text{CoM}}=\text{CoM}+\frac{v_{\text{CoM}}-v_{\text{treadmill}}}{\sqrt{g/l}}$$

CoM is the anterior-posterior center of mass position, *v*_CoM_ its velocity, *v*_treadmill_ the treadmill belt speed, *g* the gravitational acceleration, and *l* the effective leg length. In our implementation, leg length was approximated as the distance between the center of mass and the heel marker at heel strike. The MoS was then determined as the distance between the anterior boundary of the BoS at heel strike and the *x*_CoM_. For all steps, the toe markers of the plug-in gait full-body model were used to define the anterior and posterior boundaries of the BoS. This consistent definition was chosen to simplify event detection across leading and trailing limbs and to ensure uniform processing of all gait phases.


$$\text{MoS}={\text{BoS}}_{\text{boundary}}-X_{\text{CoM}}$$

Statistical significance was assessed using the nonparametric paired Wilcoxon test to analyze differences between steps, including the step immediately before the perturbation, the perturbed step, and the subsequent 3 recovery steps.

The initial study protocol aimed to use experimentally induced falls as the primary outcome. Falls were defined as harness loads of at least 30% of body weight. Post hoc inspection of the load-cell data (for methodological verification) showed that brief peaks above this threshold occurred 9 times across all perturbation trials. However, none of these events represented a visible loss of balance with full suspension in the harness, and participants were able to continue walking without interruption. Because these short, isolated peaks were not reproducible and did not meet the practical definition of an observable fall, no actual falls were analyzed. Consequently, fall-risk stratification was conducted using the FRAT-up tool as a proxy measure. To calculate the risk of falling, we used an updated version of the FRAT-up, a web-based application designed to assess fall risk in community-dwelling older adults. The original tool, referenced in [27], has been modified, and in this unpublished updated version, 2 changes were implemented: the short FES-I replaced the fear of falling assessment by Deshpande et al [36], and the 4-meter walking test was used instead of the TUG test. The updated version was chosen because it incorporated clinical and functional assessments collected in this study and thus allowed for better integration with our dataset. The FRAT-up tool was populated with the collected data, including gender, age, fall history, use of walking aids (yes or no), Parkinson disease (yes or no), stroke (yes or no), diabetes (yes or no), time for the 4-meter walking test, and the score from the short FES-I. Variables not directly assessed in our protocol—such as other comorbidities or medications—were left blank and subsequently populated according to FRAT-up guidance using literature-based prevalence estimates for community-dwelling older adults. These default or imputed values ensure completeness of the FRAT-up risk calculation even when certain data are missing. This approach is consistent with the methodology described by Palumbo et al [27]. To distinguish between high and low fall risk, the participants were divided into tertiles. Tertile classification was applied only as an exploratory approach to illustrate potential differences within the small sample. This division resulted in subgroups with lower, medium, and higher fall risk within the overall group.

To explore the ability of MoS measures to distinguish between these subgroups for the tripping scenario, receiver operating characteristic analyses were performed, providing descriptive estimates of discriminative ability. The receiver operating characteristic analyses focused on identifying the area under the curve (AUC) to determine the model’s ability to discriminate between different fall risk categories. Three combinations were used to group the FRAT-up tertiles. Variant 1 considers only the upper and lower tertiles. In the other 2 variants, the middle tertile is combined once with the lower tertile and once with the upper tertile. For all 3 variants, the MoS values for the perturbed step, as well as the subsequent 2 recovery steps, were analyzed. The AUC was also calculated for unperturbed walking in all 3 variants.

All statistical analyses were performed using R (version 4.4.1; R Foundation for Statistical Computing).

### Reporting Guideline

The reporting of this study followed the CONSORT (Consolidated Standards of Reporting Trials) extension for pilot and feasibility trials (Checklist 1) [37].

## Results

### Overview

**Figure 1.**
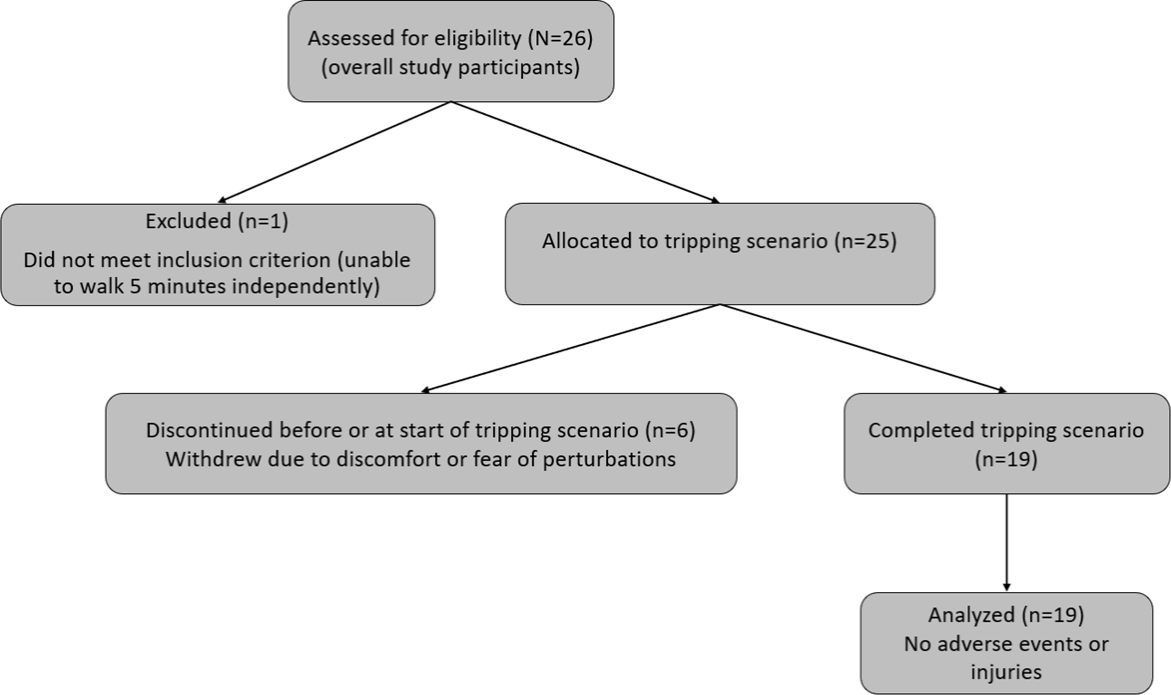
CONSORT (Consolidated Standards of Reporting Trials) flow diagram showing participant inclusion, discontinuation, and analysis in the tripping scenario of the experimental pilot study.

Figure 1 shows the CONSORT flow of participants through the tripping protocol. Nineteen of the 25 recruited subjects completed the tripping protocol. Six participants who discontinued the protocol expressed fear of falling although they were secured by a safety harness. No adverse events or injuries were observed. Table 1 provides a detailed description of the participants involved in the tripping scenario, with a mean age of 71 (SD 3.67; range 63-76) years, including 7 (37%) women.

On average, participants had 2 (SD 1.65) comorbidities and a mean short FES-I score of 8 (SD 2.27), with scores ranging from 7 to 16. The average SPPB score was 11 (SD 0.96), and the mean time for the TUG test was 8.03 (SD 1.78) seconds, indicating a relatively fit study population. Due to the fact that only 1 (5%) participant had a TUG score of more than 12 seconds, the planned classification into fit and prefrail groups was not implemented. Additionally, only 2 (10%) participants had fallen in the last 12 months. Mean score of the FRAT-up tool was 23.6 (SD 4.17).

**Table 1.** Baseline characteristics of community-dwelling older adults included in the experimental pilot study of tripping perturbations in a gait laboratory (N=19).

Characteristics	Mean (SD)	Min-Max
Age (y)	71 (3.67)	63‐76
Body height (cm)	173 (8.57)	153‐185
Body weight (kg)	81 (18.6)	49‐116
Comorbidities (0‐18)	2 (1.65)	0‐6
Short FES-I^a^ (7-28)	8 (2.27)	7‐16
SPPB^b^ (0‐12)	11 (0.96)	9‐12
TUG^c^ (s)	8.03 (1.78)	5.51‐12.78
Faller (≥1 fall in the last 12 mo)	0 (0.32)	0‐2
Score of FRAT-up^d^	23.6 (4.17)	20.4‐33.3
Slow treadmill speed (m s^–1^)	0.57 (0.17)	0.3‐0.9
Fast treadmill speed (m s^–1^)	0.76 (0.23)	0.4‐1.2

a FES-I: Falls Efficacy Scale–International.

b SPPB: Short Physical Performance Battery.

c TUG: Timed Up-and-Go Test.

d FRAT-up: Fall Risk Assessment Tool for community-dwelling older people.

### Evaluation MoS

**Figure 2.**
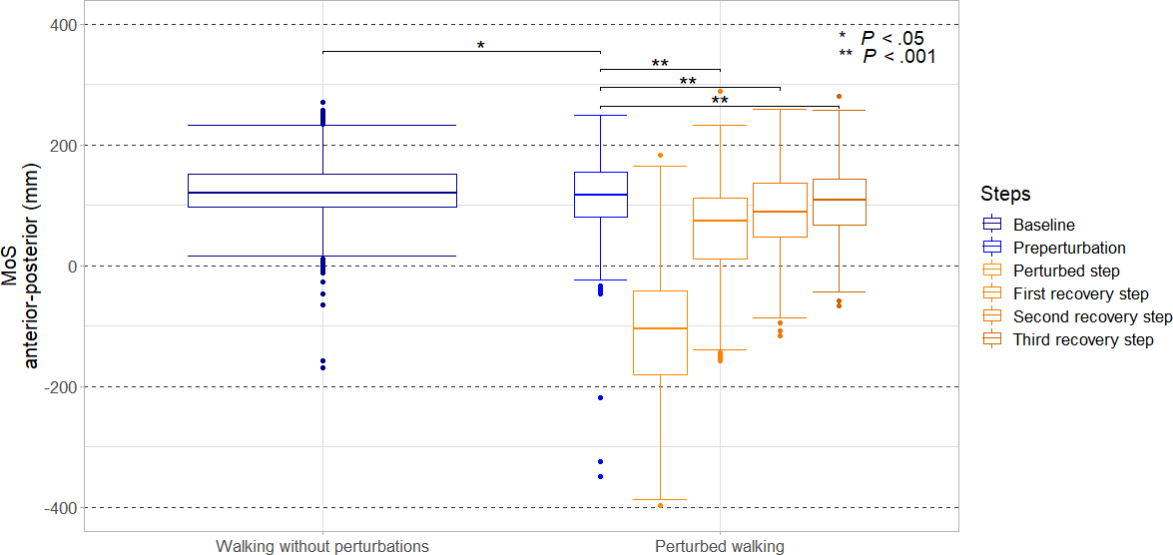
Distribution of margin of stability (MoS) values in the anterior–posterior direction during unperturbed walking as well as perturbed walking with subsequent recovery steps in community-dwelling older adults (N=19). Boxplots show preperturbation, perturbed step, and recovery steps 1‐3. Asterisks indicate significant differences between conditions (**P<.001 and * *P<.05*). Experimental pilot study conducted at the Robert Bosch Hospital gait laboratory, Stuttgart, Germany.

Figure 2 shows the steps during normal walking at the baseline measurement after the participants have acclimated to the treadmill. Furthermore, as an additional reference, the last step before the perturbation was presented. It can be observed that the median values of the MoS for unperturbed walking at baseline (median 118.85, IQR 96.88-151.50 mm) and the steps immediately preceding the perturbation (median 114, IQR 81.20-155.20 mm) differed significantly (*P*=.018). Consequently, in the following analysis, the MoS values from the last steps before the perturbation were used for comparison with the values for the perturbed step and the subsequent recovery steps 1 to 3, which are also shown in Figure 2. All conditions are presented in the same figure to identify the most affected steps for further analyses. The disturbed step showed the greatest difference to the reference values, with a median MoS of −106.05 (IQR –181.40 to   –41.50) mm, making it the only step with a median below zero. The difference between the preperturbation step and the perturbed step was highly significant (*P*<.001), underscoring the disruptive effect of the perturbation on dynamic stability. The first recovery step displayed a median MoS of 74.45 (IQR 11.90-112.80) mm, which is higher than the median value of the perturbed steps, but still significantly lower than the preperturbation step (*P*<.001). The second recovery step showed further stabilization, with a median MoS of 88.45 (IQR 47.50-137.80) mm, while the third recovery step exhibited a median MoS of 108.8 (IQR 67.10-144.18) mm. Both the second and third recovery steps demonstrated highly significant differences compared to the preperturbation step (both *P*<.001). Notably, the third recovery step approached the median MoS level of the median value of the preperturbation step. In the following analysis, the perturbed step as well as the recovery steps 1 to 3 were included, as they differed significantly from the reference (preperturbation step) and demonstrated the significant impact of the perturbation on the walking stability.

### Evaluation of Different Conditions and Step Selection

**Figure 3.**
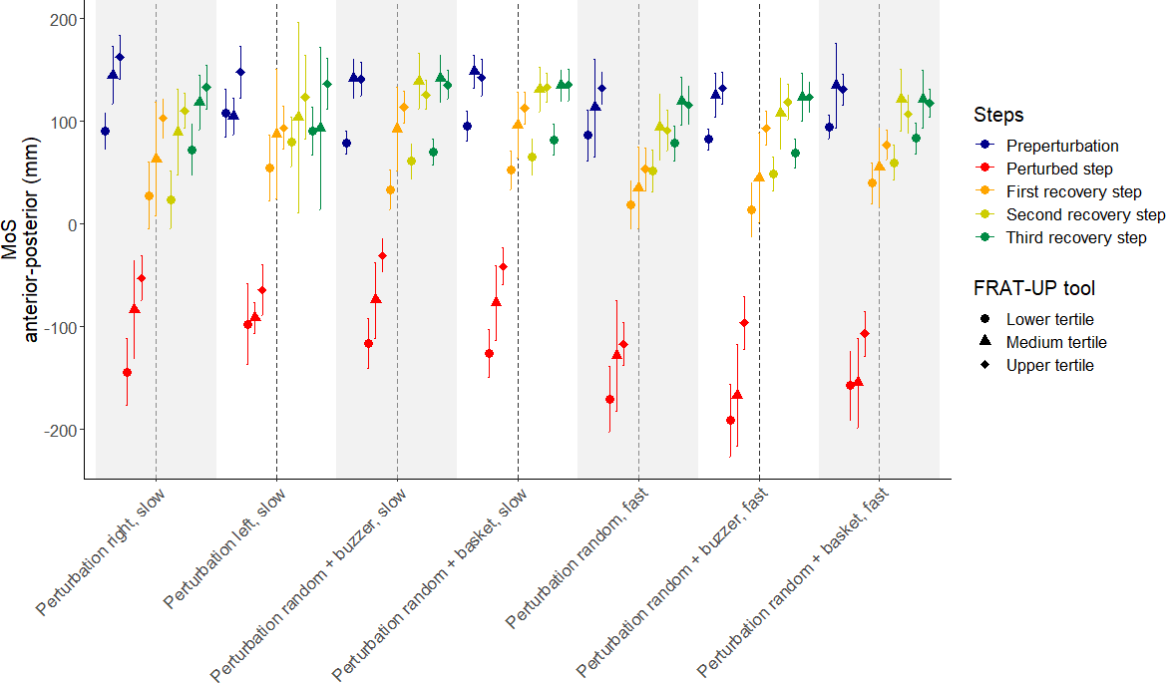
Mean values and 95% CIs of the margin of stability (MoS) in the anterior–posterior direction across different walking and perturbation conditions in community-dwelling older adults (N=19). The x-axis depicts the various perturbation conditions (slow or fast walking, series or random perturbations, and with or without additional tasks), and the y-axis shows MoS values (mm). Colors indicate the chronological steps (preperturbation, perturbed step, and recovery steps 1‐3), while symbols denote Fall Risk Assessment Tool (FRAT)-up fall-risk tertiles (circle=lower tertile or low risk, triangle=medium tertile, and diamond=upper tertile or high risk). Data were collected in an experimental pilot study at the Robert Bosch Hospital gait laboratory, Stuttgart, Germany.

Figure 3 illustrates the MoS means and 95% CIs stratified for various conditions and fall risk categories. The lower tertile (representing lower fall risk) corresponds to a FRAT score of 21.1% or less, while the upper tertile (representing higher fall risk) corresponds to a FRAT score of 25.6% or more. Table S3 in Multimedia Appendix 2 provides an overview of linear regression coefficients and their variations across steps and conditions.

The analysis focused on the MoS values for the step directly before the perturbation, the perturbed step, and the subsequent 3 recovery steps, which were further divided according to the different conditions.

The preperturbation steps revealed the fluctuating MoS values across all 3 groups. The group with the lowest fall risk consistently exhibited the smallest MoS values, while the group with the highest fall risk usually showed the largest MoS values (eg, in the “slow walking, series of perturbations right” condition, MoS values were 91 mm [lower fall risk], 145 mm [medium fall risk], and 163 mm [higher fall risk]). This trend was also evident during the baseline measurement, before the participants had experienced any perturbation. However, the middle-risk group sometimes also demonstrated higher MoS values, leading to occasional overlaps between risk groups. In the second condition (slow walking, series of perturbations left), the difference between the lower (MoS=108 mm) and the higher fall risk group (MoS=148 mm) was only 40 mm and not significant.

For the perturbed step, the MoS values decreased significantly for all groups (eg, in “fast walking, random perturbation,” values dropped to −171 mm [lower fall risk], −129 mm [medium fall risk], and −117 mm [higher fall risk]). The group with high fall risk exhibited smaller decreases in MoS compared to the other groups. However, an exception to this trend was observed in the condition “slow walking, series of perturbations left” where this difference was not as pronounced (MoS: −97 mm [low], −91 mm [medium], and −64 mm [high]).

The subsequent 3 recovery steps highlighted how participants coped with balance disturbances and their ability to regain equilibrium. Within these steps, MoS values gradually increased across all groups, with the lower fall risk group still showing the lowest values. For example, in “fast walking, random perturbations + memory buzzer,” MoS values recovered to 14 mm (lower fall risk), 45 mm (medium fall risk), and 93 mm (higher fall risk) in recovery step 1, and further increased to 49 mm (lower fall risk), 108 mm (medium fall risk), and 119 mm (higher fall risk) in recovery step 2.

Recovery steps 1 and 2 exhibited stable differences between the groups, with the lower fall risk group maintaining lower MoS values compared to the medium and higher fall risk groups. This trend was consistent across most conditions. In recovery step 3, the average rate of change in MoS decreased, and the separation between the groups became smaller.

The condition “slow walking, series of perturbations left” discriminated the least between the subgroups with lower and higher fall risk, with overlapping CIs. Similar results can be seen regarding the condition “fast walking, random perturbations.” Significant differences between the lower and higher fall-risk subgroups could be observed in all other conditions. Furthermore, conditions involving fast walking with an additional task, particularly with the use of the memory buzzer, highlighted the largest differences in MoS values between the groups during the perturbed and recovery steps (eg, perturbed step: −191 mm [lower fall risk], −167 mm [medium fall risk], −96 mm [higher fall risk]; recovery step 2: 49 mm [lower fall risk], 108 mm [medium fall risk], 119 mm [higher fall risk]).

### Evaluation Outcomes

Table 2 presents the results for the AUC values for different analysis variants. Overall, variant 1 (*T*_low_ vs *T*_high_, where *T*_low_ represents the lower tertile=lower fall risk and *T*_high_ represents the upper tertile=higher fall risk) yields the best results for unperturbed walking and also for the perturbation scenarios compared to the other two, with AUCs of 76.7% and 86.2%, respectively. However, variant 1 did not include all subjects. Therefore, variants 2 (*T*_low_ versus *T*_mid_+ *T*_high_) and 3 (*T*_low_+ *T*_mid_ vs *T*_high_) were particularly compared.

**Table 2.** Area under the curve (AUC) for margin of stability (MoS) in differentiating between fall-risk tertiles in community-dwelling older adults (N=19). Experimental pilot study conducted at the Robert Bosch Hospital gait laboratory, Stuttgart, Germany. Comparisons were made between the lower tertile (*T*_low_, low fall risk) versus the higher tertile (*T*_high_, high fall risk), *T*_low_ versus the combined middle and higher tertiles (*T*_mid_+ *T*_high_), and the combined lower and middle tertiles (*T*_low_+ *T*_mid_) versus *T*_high_. Conditions include unperturbed walking, slow or fast walking with series or random perturbations, and perturbations combined with cognitive (buzzer) or motor (basket carrying) tasks. Values are for perturbed walking during the perturbed step, recovery step 1, and recovery step 2. Superscript letters indicate best and worst values within analyses and best discriminative performance across key comparisons.

	*T*_low_ versus *T*_high_ (AUC; %)	*T*_low_ versus *T*_mid_+ *T*_high_ (AUC; %)	*T*_low_+ *T*_mid_ vs *T*_high_ (AUC; %)
Walking without perturbation (all steps)	76.7	76.5^a^	69.3
Slow walking, series of perturbations right	82.2, 78.4^b^, 86.2^c^	78.4, 73.3^b^, 82.3^a^^,^^c^	75.1, 72.8^b^, 77.7^c^
Slow walking, series of perturbations left	60.4, 59.2^b^, 69.4^c^	58.8, 58.7^b^, 68.9^c^	60.8, 58.7^b^, 69.2^a^^,^^c^
Slow walking, random perturbations+ buzzer	78.8^b^, 81.8, 80.2^c^	72.3^b^, 77.5, 80.1^a^^,^^c^	75.6^c^, 74.0, 69.5^b^
Slow walking, random perturbations+ basket	79.9, 76.4^b^, 82.2^c^	74.7, 72.6^b^, 81.2^a^^,^^c^	74.3^c^, 69.6^b^, 72.0
Fast walking, random perturbations	66.2^c^, 64.0^b^, 64.9	63.5, 60.6^b^, 64.4^a^^,^^c^	63.1^c^, 61.1, 59.1^b^
Fast walking, random perturbations+ buzzer	71.7^b^, 76.9, 80.7^c^	66.0^b^, 70.1, 78.4^a^^,^^c^	71.7^c^, 74.0, 71.5^b^
Fast walking, random perturbations+ basket	63.4^b^, 68.3, 72.7^c^	58.5^b^, 63.1, 73.3^a^^,^^c^	64.7^c^, 64.6, 62.8^b^

a Best discriminative performance for the two key comparisons (*T*_low_ vs *T*_mid_+*T*_high_ and *T*_low_+*T*_mid_ vs *T*_high_).

b Worst value within that variant.

c Best value within a given analysis.

Variant 1 already showed that the second recovery step during perturbation scenarios predominantly demonstrated the best discriminative ability. The same trend was noticeable in variant 2, where the lower and middle tertiles were combined into 1 group. Across all scenarios, the discriminative ability was best during the second recovery step. Baseline walking exhibited an acceptable performance with an AUC of 76.5%. However, the best results were observed in the condition “slow walking, series of perturbations right” (82.3%), followed by “slow walking, random perturbations + buzzer” (80.1%) as well as slow walking with random perturbations and carrying a basket (81.2%). Generally, in all 3 variants, analyses with slow walking speed showed stronger discriminative ability than the others, except for the series of perturbations to the left condition.

**Figure 4.**
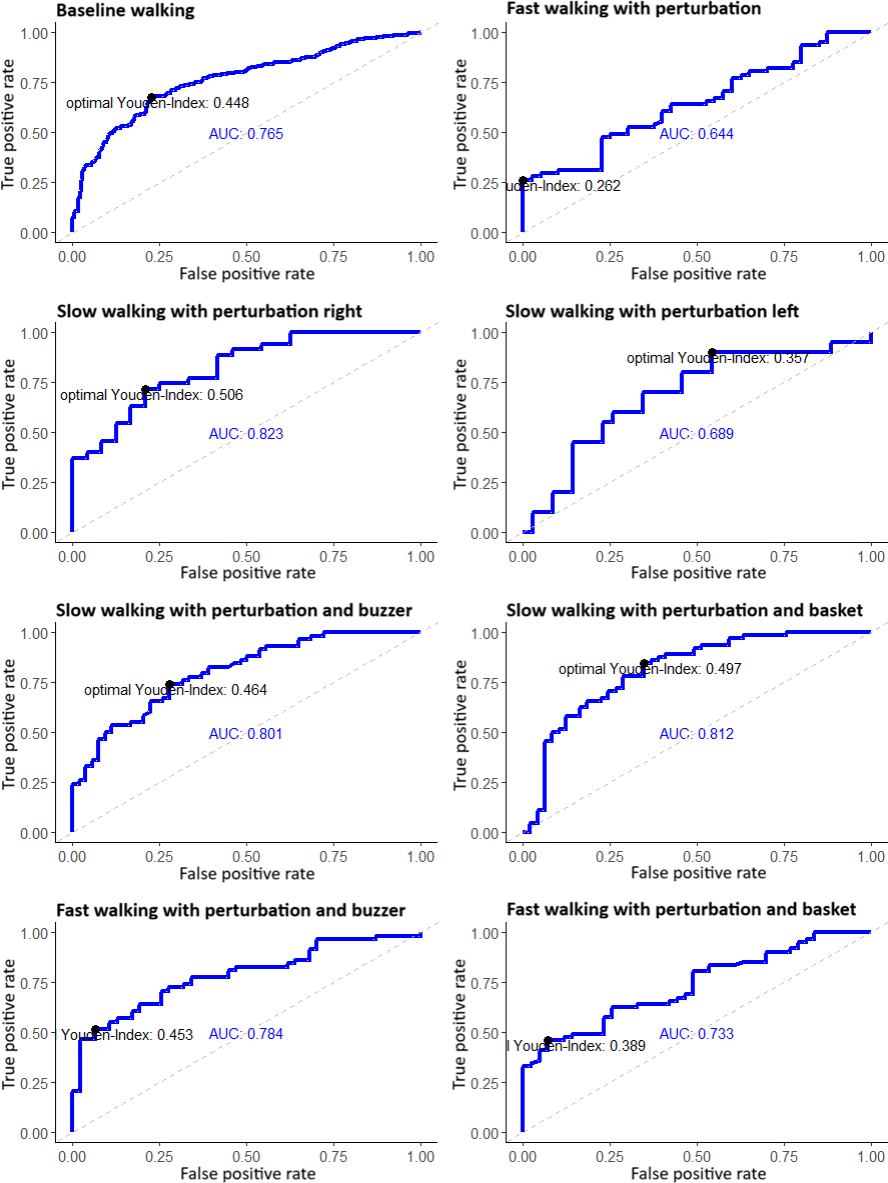
Receiver operating characteristic (ROC) curves showing the area under the curve (AUC) for margin of stability (MoS) values in the second recovery step across different walking and perturbation conditions in community-dwelling older adults (N=19). Analyses differentiate between the lower Fall Risk Assessment Tool (FRAT)-up tertile (representing lower fall risk) and the combined middle and upper tertiles (representing higher fall risk). Panels illustrate results for baseline walking as well as slow and fast walking with perturbations, with or without additional cognitive (buzzer) or motor (basket carrying) tasks. Experimental pilot study conducted at the Robert Bosch Hospital gait laboratory, Stuttgart, Germany.

In variant 3, the analysis showed the best results during the perturbed step. However, the second recovery step exhibited even better results compared to the first recovery step. When comparing variants 2 and 3, variant 2 outperformed the other one, except for the condition “slow walking, series of perturbations left.” In this case, the discriminative ability was best during the second recovery step. The AUC values for this variant are depicted in Figure 4.

## Discussion

### Principal Findings

This pilot study examined the feasibility of a tripping paradigm and its exploratory association with fall-risk indices in community-dwelling older adults. In terms of feasibility, the study revealed important aspects. Approximately one quarter of participants discontinued before or at the start of the tripping scenario because of discomfort or fear of perturbations. While this confirmed the overall safety of the protocol, it also highlighted that the tripping paradigm may be challenging for older adults with lower confidence or higher frailty levels. These reactions underscore that procedural refinements, such as extended familiarization, gradual perturbation intensities, or shorter testing sessions, may be necessary to enhance tolerability and extend feasibility to frailer populations in future studies. Importantly, the fact that several participants discontinued before or during the tripping scenario highlights a critical feasibility limitation of the current setup. Even among relatively fit older adults, the protocol was perceived as challenging, suggesting that its direct application in frailer populations may not be feasible without substantial procedural adaptation. The study was originally designed to experimentally induce near-falls or loss-of-balance events to assess stability thresholds. However, no participants reached the predefined fall criterion during this scenario, likely because those most prone to losing balance discontinued participation due to discomfort or fear. This indicates that the paradigm may require modification to safely elicit critical balance losses in future, more heterogeneous samples. In this pilot, heterogeneity was not the primary aim; the focus was to first test procedural feasibility and ensure participant safety. Although objective safety was maintained at all times, perceived safety concerns led several participants to discontinue, highlighting that future adaptations should also aim to enhance participants’ sense of security when applying the paradigm to frailer populations. Beyond feasibility, three key findings emerged. First, group differentiation was already apparent during baseline walking, suggesting that steady-state gait measures may capture relevant aspects of fall risk even without perturbations. Second, the second recovery step consistently provided the clearest separation between risk strata, highlighting its potential value as a marker of balance recovery capacity. Third, the tertile-based stratification yielded counterintuitive patterns, with higher fall-risk groups sometimes exhibiting larger MoS values, which we interpret in light of compensatory strategies and risk perception. These findings are discussed below in relation to previous research.

Overall, the results highlight the significant impact of perturbations on dynamic stability, as measured by the MoS. Perturbations caused marked disruptions, with the perturbed step showing the largest deviation, evidenced by a median MoS below zero. Recovery steps revealed a gradual stabilization process. While the second recovery step may play a role in recovery dynamics, its specific contribution to fall risk group differentiation remains underexplored in the existing literature. Taken together, this supports the view that recovery dynamics, rather than the perturbed step itself, may provide more nuanced insight into fall-risk differentiation. Additionally, broader research emphasizes the use of dynamic assessments, such as those highlighted by Herssens et al [15], in identifying stability deficits that may relate to fall risk.

Interestingly, differentiation between fall-risk groups was already apparent during baseline walking, even without perturbations. Gait variability in steady-state walking, as emphasized by Menant et al [38], underscores the potential of baseline gait measures for identifying fall risk in diverse populations. While Kazanski et al [12] primarily focused on perturbation-induced variability, their findings align with this perspective. Importantly, recent research has shown that MoS can be reliably estimated using wearable inertial sensors, enabling its use as a low-resource screening tool. Akiyama et al [39] demonstrated that MoS can be classified with over 90% accuracy in both forward and lateral directions using only inertial measurement unit acceleration data, even under gait asymmetry conditions. Similarly, Guaitolini et al [40] validated a mobile, magneto-inertial sensor-based approach to assess MoS with high accuracy in daily living conditions. Baseline walking MoS assessments could serve as a low-resource, accessible screening tool in the future, particularly in settings without advanced perturbation setups. Delbaere et al [41] highlighted that baseline measures are particularly effective in identifying intrinsic stability differences. Their findings also underscore that the disparity between perceived and physiological fall risk significantly influences fall likelihood, reinforcing the importance of considering psychological factors such as overconfidence and fear of falling in screening and prevention strategies.

The tertile classification provides valuable insights into fall risk stratification but also reveals challenges, particularly regarding the middle tertile, where overlaps with low- and high-risk groups complicate interpretation. This limitation likely reflects the high mobility and narrow functional range of the present cohort, reducing between-group differentiation. Accordingly, these results should be considered exploratory.

As outlined in the introduction, prior studies [19-23] generally found that lower MoS is associated with higher fall risk. In contrast, in our exploratory analysis, the low fall-risk group exhibited lower MoS values compared to the high fall-risk group across all conditions. While higher MoS values typically indicate greater stability, this counterintuitive result may be related to differences in risk perception, as suggested by Delbaere et al [41] and Young and Williams [42]. However, these interpretations remain speculative since the cited studies did not involve perturbation paradigms, which primarily elicit automatic postural responses. This unexpected direction of effects also raises questions about how well the present paradigm captures stability differences related to fall risk. Although MoS differences between risk strata were evident, their direction was contrary to theoretical expectations. Further validation in larger, more heterogeneous samples with prospective fall data is needed to challenge this finding. Robinovitch et al [18] analyzed real-life falls in long-term care settings and showed that overconfidence during routine activities, such as walking or standing quietly, often led to incorrect weight shifts, a primary cause of falls. Taken together, these findings suggest that both behavioral and biomechanical factors may contribute to fall risk. Future studies should assess confidence levels alongside biomechanical measures to better capture these dynamics.

The analysis of recovery steps confirmed the value of the second recovery step in differentiating fall risk. This step demonstrated consistent increases in MoS and clear differentiation between tertiles, highlighting its significance as a critical phase for assessing fall risk recovery dynamics. In contrast, the third recovery step demonstrated smaller MoS changes and reduced separation between groups, limiting its relevance. These findings align with Song et al [43], who emphasized the importance of early recovery phases in understanding and assessing stability dynamics. One possible explanation for its relevance is that the second recovery step marks the crucial transition where the CoM is brought back into the base of support. During this phase, the remaining destabilizing forces are compensated for, and the body is repositioned, enabling effective balance recovery and overall stabilization. Individuals at high risk of falling might have greater difficulty effectively returning the CoM to the base of support during this step, potentially resulting in poorer balance recovery. As such, analyzing the second recovery step could provide valuable exploratory insights for distinguishing between individuals with higher and lower fall risk, although validation in larger and frailer cohorts is required.

Perturbation response varied across experimental conditions. Slow walking conditions, especially those involving secondary tasks such as carrying a basket or using the memory buzzer, yielded the clearest tertile differentiation, with AUC values reaching 0.823. In contrast, fast walking with random perturbations showed lower AUC values. This is consistent with Song et al [44], who noted that higher walking speeds reduce MoS variability across subjects. There could also be a ceiling effect for stability at high walking speed. Together, these findings emphasize the need to test perturbation paradigms across a range of conditions to identify which are most sensitive for differentiating fall risk.

It was observed that right and left leg responses might differ, but asymmetries in perturbation response were not analyzed separately due to limited data. However, previous studies suggest this could provide valuable insights. Liu and Finley [45] and Kozlowska et al [46] found that dominant legs contribute more to controlling gait deviations, potentially affecting recovery strategies. Including assessment and analysis of effects by limb dominance might further improve the results.

### Limitations

This study has several limitations. The small sample size, with only 19 participants completing the tripping protocol out of the initial 25 recruited, limited the generalizability of the findings and reduced statistical power for subgroup analyses. The study population, characterized by high mobility and good balance (as indicated by TUG and SPPB scores), further constrained variability and reduced differentiation between risk groups. No actual falls were observed during testing, partly due to participants’ heightened awareness of upcoming perturbations and related discomfort, which also contributed to several dropouts. Because of the absence of fall events, the FRAT-up tool was used as an alternative, exploratory stratification measure. While the FRAT-up tool demonstrated moderate predictive accuracy (AUC=0.65), it lacked a clear cutoff for classification. Additionally, the updated version used in this study has not yet been independently validated, and its psychometric properties remain uncertain.

Another limitation is that interindividual differences in self-selected treadmill speeds may have influenced MoS values, as walking speed directly enters the *x*_CoM_ calculation. We chose self-selected speed to ensure ecological validity and participant safety, and both slow and fast conditions were defined relative to the baseline. Nevertheless, between-subject comparisons should be interpreted with caution.

Further sources of bias must also be considered. These include possible measurement errors due to transient marker occlusions, manual corrections of gait events, and assumptions in the Plug-in Gait model (eg, estimation of CoM position and effective leg length). Variability introduced by individualized treadmill speeds and the nonparametric statistical methods applied in a small sample further limits robustness. Moreover, certain clinical and environmental risk factors (eg, medication use and home hazards) were not captured in this protocol but may significantly influence fall risk in real-world settings.

The treadmill-based setup, while offering practical replication of real-world scenarios, introduced its own set of challenges. The use of a safety harness, essential for participant safety, may have reduced the perceived risk and potentially influenced behavior during testing.

### Conclusions

This pilot and feasibility study provides preliminary evidence that MoS measures differ between fall risk categories in community-dwelling older adults. Baseline walking already showed some group separation, and perturbation trials yielded stronger exploratory associations, particularly in the second recovery step. Slow walking conditions appeared especially sensitive in this small sample. However, these findings must be interpreted cautiously given the small, relatively fit cohort, the exploratory tertile-based stratification, and potential methodological biases. The results should therefore be seen as hypothesis-generating rather than conclusive. Future research with larger and frailer populations, as well as additional fall scenarios, is needed to confirm these preliminary patterns and to determine whether MoS can ultimately serve as a clinically useful measure of fall risk. The fact that no participants reached the predefined fall threshold in this scenario further highlights the feasibility limitations of the current multitask-based setup. There is a need to refine the perturbation paradigms to elicit loss of balance in a safe yet ecologically valid manner, particularly when extending such protocols to more vulnerable populations.

## Supplementary material

10.2196/74418 Multimedia Appendix 1 Schematic illustration of the treadmill-based tripping experiment setup. Participants walked on a treadmill while wearing a safety harness attached to an overhead rail. Straps were fixed to both heels with cords extending backward to induce tripping perturbations.

10.2196/74418 Multimedia Appendix 2 Regression coefficients of the margin of stability (in mm) across different perturbation conditions and steps in community-dwelling older adults (N=19). Values represent regression coefficients stratified by fall-risk tertiles according to the Fall Risk Assessment Tool for community-dwelling older people index (low risk, medium risk, and high risk). Conditions include slow and fast walking with series or random perturbations, as well as perturbations combined with a concurrent cognitive task (memory buzzer) or a motor task (basket carrying). Steps are presented separately for preperturbation, perturbed step, and recovery steps 1-3. Experimental pilot study conducted at the Robert Bosch Hospital gait laboratory, Stuttgart, Germany.

10.2196/74418 Checklist 1 CONSORT checklist for pilot or feasibility trial.

## Abbreviations

AUC: area under the curve
BoS: base of support
CoM: center of mass
CONSORT: Consolidated Standards of Reporting Trials
FES-I: Falls Efficacy Scale–International
FRAT-up: Fall Risk Assessment Tool for community-dwelling older people
MoS: margin of stability
ROC: receiver operating characteristic
SPPB: Short Physical Performance Battery
TUG test: Timed Up-and-Go test
