# Supplementary figures and images for: Association of Fall-Risk Factors and Margin of Stability While Tripping in Community-Dwelling Older Adults: Experimental Pilot Study
Source: JMIR Form Res. 2026 Feb 5;10:e74418. doi: 10.2196/74418 (PMC12875566; doi:10.2196/74418)

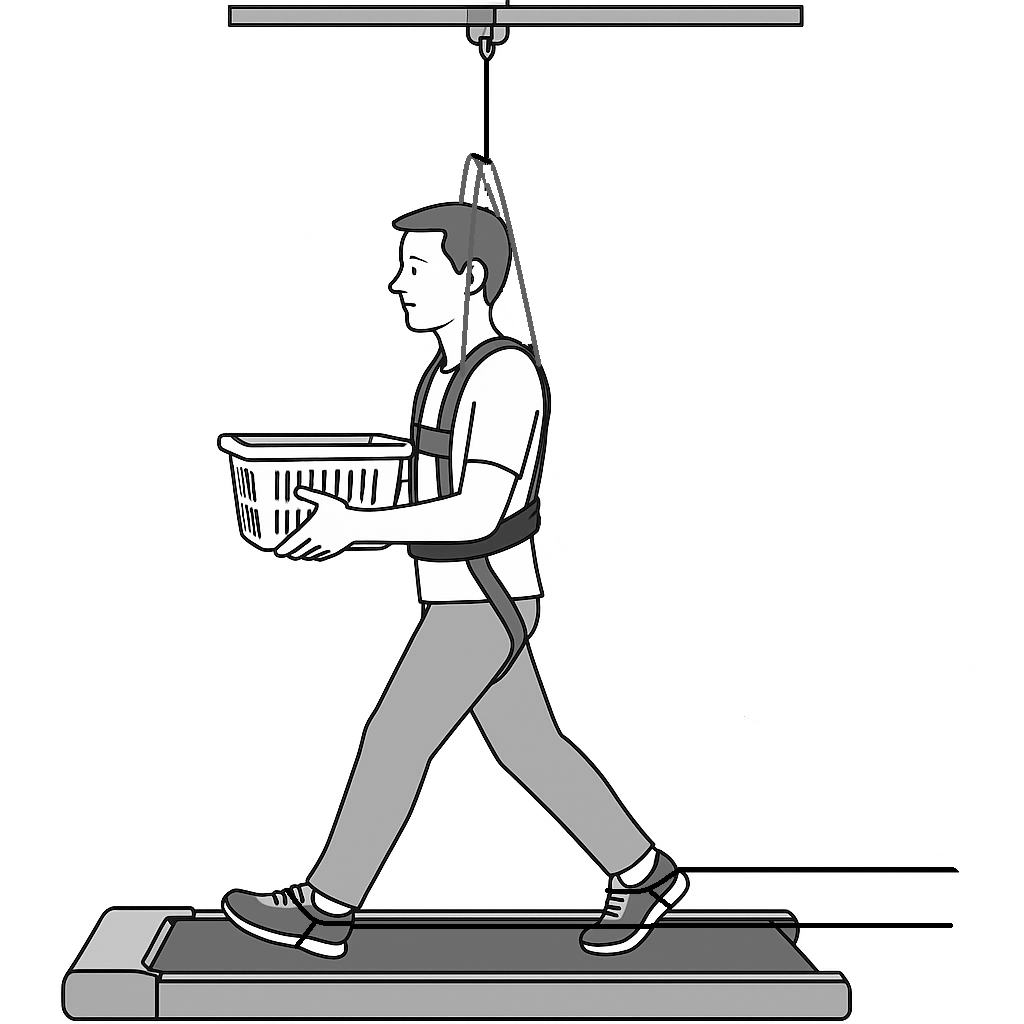

Supplement: Multimedia Appendix 1 [file formative-v10-e74418-s001.png]
