# Supplementary material for: Association of Fall-Risk Factors and Margin of Stability While Tripping in Community-Dwelling Older Adults: Experimental Pilot Study
Source: JMIR Form Res. 2026 Feb 5;10:e74418. doi: 10.2196/74418 (PMC12875566; doi:10.2196/74418)
Supplement: Multimedia Appendix 2 [file formative-v10-e74418-s002.docx]

Table 3: Regression coefficients of the margin of stability (MoS, in mm) across different perturbation conditions and steps in community-dwelling older adults (n = 19). Values represent regression coefficients stratified by fall-risk tertiles according to the FRAT-up index (low, medium, high risk). Conditions include slow and fast walking with series or random perturbations, as well as perturbations combined with a concurrent cognitive task (memory buzzer) or a motor task (basket carrying). Steps are presented separately for pre-perturbation, perturbed step, and recovery steps 1–3. Experimental pilot study conducted at the Robert Bosch Hospital gait laboratory, Stuttgart, Germany.

| **Step** | **Condition** | **low**  **fall risk** | **medium**  **fall risk** | **high**  **fall risk** |
| --- | --- | --- | --- | --- |
| **Pre-perturbation** | Slow walking,  series of perturbations right | 91 | 54 | 72 |
|  | Slow walking,  series of perturbations left | 108 | -4 | 40 |
|  | Slow walking, random perturbations + memory buzzer | 79 | 62 | 62 |
|  | Slow walking, random perturbations + basket | 96 | 53 | 47 |
|  | Fast walking, random perturbations | 87 | 27 | 46 |
|  | Fast walking, random perturbations + memory buzzer | 83 | 43 | 50 |
|  | Fast walking, random perturbations + basket | 94 | 40 | 37 |
| **Perturbed step** | Slow walking, series of perturbations right | -144 | 61 | 92 |
|  | Slow walking, series of perturbations left | -97 | 6 | 33 |
|  | Slow walking, random perturbations + memory buzzer | -116 | 42 | 86 |
|  | Slow walking, random perturbations + basket | -126 | 49 | 85 |
|  | Fast walking, random perturbations | -171 | 42 | 54 |
|  | Fast walking, random perturbations + memory buzzer | -191 | 24 | 95 |
|  | Fast walking, random perturbations + basket | -157 | 3 | 51 |
| **First recovery step** | Slow walking, series of perturbations right | 28 | 36 | 75 |
|  | Slow walking, series of perturbations left | 54 | 33 | 39 |
|  | Slow walking, random perturbations + memory buzzer | 33 | 60 | 81 |
|  | Slow walking, random perturbations + basket | 52 | 44 | 61 |
|  | Fast walking, random perturbations | 19 | 16 | 35 |
|  | Fast walking, random perturbations + memory buzzer | 14 | 31 | 79 |
|  | fast walking, random perturbations + Basket | 40 | 15 | 37 |
| **Second recovery step** | Slow walking, series of perturbations right | 24 | 66 | 87 |
|  | Slow walking, series of perturbations left | 80 | 24 | 44 |
|  | Slow walking, random perturbations + Memory buzzer | 61 | 78 | 65 |
|  | Slow walking, random perturbations + Basket | 65 | 66 | 68 |
|  | Fast walking, random perturbations | 52 | 43 | 39 |
|  | Fast walking, random perturbations + Memory buzzer | 49 | 59 | 70 |
|  | Fast walking, random perturbations + Basket | 60 | 61 | 47 |
| **Third recovery step** | Slow walking, series of perturbations right | 72 | 46 | 61 |
|  | Slow walking, series of perturbations left | 91 | 2 | 46 |
|  | Slow walking, random perturbations + Memory buzzer | 70 | 72 | 65 |
|  | Slow walking, random perturbations + Basket | 82 | 53 | 53 |
|  | Fast walking, random perturbations | 79 | 41 | 38 |
|  | Fast walking, random perturbations + Memory buzzer | 69 | 55 | 55 |
|  | Fast walking, random perturbations + Basket | 84 | 38 | 34 |
